# Early initiation of SGLT2 inhibitors after acute myocardial infarction

**DOI:** 10.1093/ehjcvp/pvaf020

**Published:** 2025-04-18

**Authors:** Andeas Hammer, Samuel Sossalla, Patrick Sulzgruber

**Affiliations:** Division of Cardiology, Department of Internal Medicine II, Medical University of Vienna, Weahringer Guertel 18-20, 1090 Vienna, Austria; Medical Clinic I, Cardiology and Angiology, Justus-Liebig-University, Klinikstrasse 33, 35392 Giessen, Germany; Department of Cardiology, Kerckhoff-Clinic/DZHK, Benekestrasse 2-8, 61231 Bad Nauheim, Germany; Division of Cardiology, Department of Internal Medicine II, Medical University of Vienna, Weahringer Guertel 18-20, 1090 Vienna, Austria

**Keywords:** SGLT2 inhibitors, STEMI, NSTEMI, Heart failure, Acute myocardial infarction

Over the past decade, sodium-glucose cotransporter-2 (SGLT2) inhibitors have emerged as key agents providing cardiovascular, renal, and metabolic protection in chronic conditions. Recent evidence from a large Scandinavian cohort study indicates that empagliflozin and dapagliflozin offer comparable cardiovascular and renal efficacy, with similar risks of major cardiovascular events, heart failure (HF) hospitalizations, and serious renal events, as well as no significant differences in overall mortality or diabetic ketoacidosis.^1^ More recently, their potential benefits in acute settings, such as after acute myocardial infarction (AMI), have attracted increasing interest, potentially expanding the therapeutic indications for SGLT2 inhibitors in cardiovascular care.^2^

In this regard, the EMMY trial demonstrated that empagliflozin (10 mg daily) in patients with large AMI significantly reduced n-terminal pro-b-type natriuretic peptide (NT-proBNP) levels (−15% vs. placebo) within 12 weeks and improved with left ventricular ejection fraction (LVEF), diastolic function, and ventricular remodelling. Conducted in 476 patients randomized within 72 h post-percutaneous coronary intervention (PCI), the study suggested a potentially faster cardiac recovery with SGLT2 inhibition.^3^ Nevertheless, the study did not allow any conclusions regarding clinical endpoints, as it was not powered for this question.

**Figure 1:**
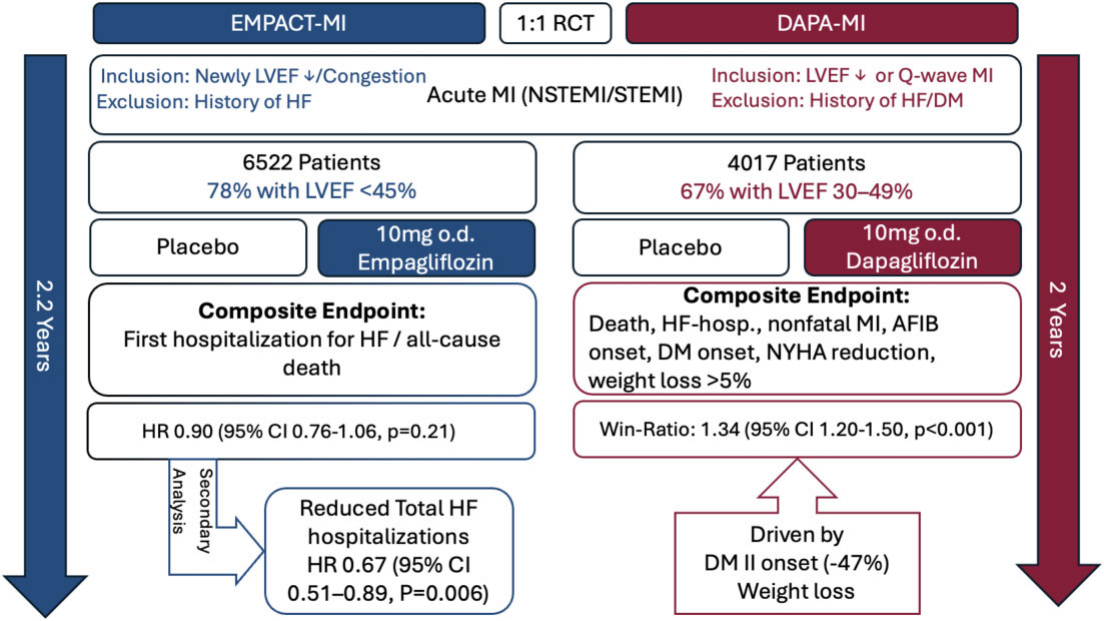
Overview of the EMPACT-MI and DAPA-MI trials. Legend: A brief overview of the DAPA-MI and EMPACT-MI trials. Abbreviations: AFIB, atrial fibrillation; CI, confidence interval; DM, diabetes mellitus; HF, heart failure; HR, hazard ratio; LVEF, left ventricular ejection fraction; MI, myocardial infarction.

The DAPA-MI trial investigated the effect of dapagliflozin on cardiometabolic outcomes in patients with AMI without a history of diabetes mellitus (DM) or HF.^4^ This randomized trial included 4017 clinically stable patients, aged 63 on average (20% female), who received either dapagliflozin 10 mg daily or placebo alongside standard care for 24 months. Participants were enrolled within 10 days of AMI (NSTEMI or STEMI), with most having impaired left ventricular function (67% with LVEF 30–49%).^4^ The trial reported a positive composite primary outcome, which included death, HF hospitalization, non-fatal MI, atrial fibrillation/flutter events, new-onset DM, NYHA class changes, and weight loss, with a win ratio of 1.34 [95% confidence interval (CI) 1.20–1.50, *P* < 0.001].^4^ This win-ratio outcome was primarily driven by a 47% reduction in new-onset DM [hazard ratio (HR) 0.53; 95% CI 0.36–0.77] and an average weight loss of 1.65 kg over 24 months in the treatment group.^4^ Notably, although ∼75% of participants had reduced LVEF, effects in all cardiovascular endpoints, such as cardiovascular death or hospitalization for HF, did not differ between the treatment groups. These results suggest that while dapagliflozin offers notable cardiometabolic benefits, particularly in reducing DM risk and weight, its impact on cardiovascular outcomes after AMI might be limited.

Similarly, the EMPACT-MI trial evaluated the impact of early initiation of empagliflozin in 6522 patients with AMI at high risk of HF, defined as clinical signs and symptoms of congestion that required medical therapy (57.0%), or newly reduced LVEF (78.4% with LVEF <45%).^5^ Participants were randomized to receive empagliflozin 10 mg daily or placebo within 14 days post-AMI, with a median follow-up of 17.9 months.^5^ Despite not meeting the primary endpoint (all-cause mortality, or first HF hospitalization), empagliflozin showed consistent reduction in HF hospitalizations across the entire range of LVEF and congestion profiles.^5^

In a pre-specified secondary analysis of EMPACT-MI, HF outcomes were evaluated between treatment groups.^6^ Over a median follow-up of 17.9 months, empagliflozin significantly reduced the risk of first HF hospitalization (HR 0.77; 95% CI 0.60–0.99; *P* = 0.031) and total HF hospitalizations (HR 0.67; 95% CI 0.51–0.89; *P* = 0.006) compared with placebo. Additionally, empagliflozin lowered the risks of first HF hospitalization or HF-related death (HR 0.78; 95% CI 0.62–0.98; *P* = 0.031) and total HF hospitalizations or HF-related death (HR 0.69; 95% CI 0.51–0.93; *P* = 0.015).^6^ These benefits were consistent across subgroups, including patients with ST-elevation myocardial infarction (STEMI) or non-st-elevation myocardial infarction (NSTEMI) and those with or without DM.^6^ Interestingly, among patients discharged without diuretic therapy, those in the empagliflozin group were less likely to start diuretics other than mineralocorticoid receptor antagonist (MRAs) within 6 months compared to placebo (12.2% vs. 15.3%; HR 0.80; *P* = 0.046).^6^ Similarly, fewer empagliflozin-treated patients initiated ARNI (HR 0.73; *P* = 0.009), ACE inhibitors/angiotensin receptor blocker (ARBs)/angiotensin receptorneprilysin inhibitor (ARNI) (HR 0.75; *P* = 0.044), or MRAs (HR 0.74; *P* = 0.017) post-discharge.^6^

In conclusion, while SGLT2 inhibitors offer promising cardiometabolic and HF benefits in post-AMI patients, their long-term impact on robust outcomes such as mortality remains unclear. However, the absence of a mortality signal in both DAPA-MI and EMPACT-MI raises questions about their routine post-AMI use of SGLT2 inhibitors, especially considering cost and safety concerns. While reducing HF hospitalizations is meaningful, it remains unclear whether this justifies widespread initiation early after AMI. Nonetheless, data from the EMMY trial suggest a faster recovery after ischaemia, which may correlate with the lower HF hospitalization rate driven by empagliflozin. Moving forward, a more selective, personalized approach focusing on high-risk subgroups is most likely to prove beneficial. Until further evidence clarifies their role, clinicians should continue prioritizing established post-MI therapies with proven mortality benefits while considering SGLT2 inhibitors particularly for high-risk patients who may derive the greatest benefit.

## Data Availability

All data referenced in this article are accessible through the cited references.
